# Reliability of carotid-femoral arterial waveforms for the derivation of ultra-short term heart rate variability in injured British servicemen: An inter-rater reliability study

**DOI:** 10.1371/journal.pone.0290618

**Published:** 2023-09-01

**Authors:** Rabeea Maqsood, Ahmed Khattab, Alexander N. Bennett, Christopher J. Boos

**Affiliations:** 1 Faculty of Health and Social Sciences, Bournemouth University, Bournemouth, United Kingdom; 2 National Heart and Lung Institute, Faculty of Medicine, Imperial College London, London, United Kingdom; 3 Academic Department of Military Rehabilitation, Defence Medical Rehabilitation Centre, Stanford Hall, Loughborough, United Kingdom; 4 Department of Cardiology, University Hospital Dorset, NHS Trust, Poole, United Kingdom; University of Southern California, UNITED STATES

## Abstract

In this study, the comparative precision of carotid versus femoral arterial waveforms to measure ultra-short term heart rate variability (HRV_UST_) following traumatic injury was investigated for the first time. This was an inter-rater reliability study of 50 British servicemen (aged 23–44 years) with non-acute combat-related traumatic injury (CRTI). Paired continuous arterial waveform data for HRV_UST_ analysis, were simultaneously sampled at the carotid and femoral arterial sites (14–16 seconds) during pulse wave velocity (PWV) measurement. HRV_UST_ was reported as the root mean square of the successive differences (RMSSD). Following the determination of the superior sampling site (carotid versus femoral), the blinded inter-rater agreement in RMSSD for the preferred site was quantified using the Intra-class Correlation Coefficient (ICC) and the Bland-Altman plot. The mean age of participants was 34.06±4.88 years. The femoral site was superior to the carotid site with a significantly higher number of reliable signals obtained (Fisher’s Exact test; p<0.001). The inter-rater agreement in femoral-derived RMSSD was excellent [ICC 0.99 (95%CI: 0.994–0.997)] with a moderate level of agreement (mean difference [bias]: 0.55; 95% CI: -0.13–1.24 ms). In this study, we demonstrated that the femoral artery is a more reliable site than the carotid artery for HRV_UST_ measurement and post-trauma risk stratification following CRTI.

## Introduction

Heart rate variability (HRV) is a non-invasive measure of autonomic function and sympathetic-parasympathetic balance [1, 2]. It relies on a variety of complex mathematical computations of the cardiac inter-beat intervals (IBI). Traditional measures of HRV relate to either 5-minute (short-term) or 24-hour (long-term) electrocardiogram (ECG) recordings using the R-R interval (RRI) as a proxy for IBI. While 24-hour recordings provide a much greater source of IBI data, the 5-min epochs have also shown validity and reliability and is the gold standard short-term HRV recording period [3]. Moreover, as the 5-minute recordings are undertaken during supine rest, it is more practical and reproducible than longer recordings as they are not prone to the influence of autonomic responses to exercise, eating, and sleep [4]. Even shorter HRV analyses of under 5 minutes, known as ultra-short short-term HRV (HRV_UST_), have gained increasing clinical and research traction owing to its easier application and lower patient burden [1, 4–6].

While ECG remains the gold standard tool used to measure IBI for HRV analysis [3], the use of several other sources of IBI quantification has been validated. Examples include radial and digital plethysmography using the peripheral arterial pulse waveform, which appears to be a reliable alternative, particularly for time-domain measures of short-term HRV [7, 8]. Another method is the use of pulse waveform analysis (PWA) of the brachial and other more proximal arterial waveforms owing to their larger volume pulse for signal acquisition [8]. Of these, the use of the brachial arterial waveform has been the most practical. Cardiac IBIs calculated using sequential brachial pulse waveforms have been reported to strongly correlate with the gold standard RRI acquired using a traditional single lead ECG in both healthy adults [9] and a variety of medical conditions [8].

To date, most of the pulse waveform HRV research has tended to short-term HRV (5–10-minute recordings) using devices such as the EndoPAT^®^ (Itamar Medical Ltd Caesarea, Israel) [7], the Finapres^®^ and Finometer^®^ (Finapres Medical Systems) [10, 11] with HRV reported as the root mean square of the successive differences (RMSSD) [4–6]. However, with improvements in software technology and IBI detection algorithms, the use of HRV_UST_ as a reliable proxy for longer recordings (≥5 minutes) has emerged. Modern portable devices such as the ithlete™ (HRV Fit Ltd, Southampton, UK) and the Uscom BP^+^ device (Uscom, Sydney, NSW, Australia) have been well-validated as a reliable source of HRV_UST_ [12–14].

The use and reliability of carotid-femoral waveforms, typically recorded during pulse wave velocity (PWV) measurement have been barely examined. Traditionally, PWV is measured over the carotid and femoral region (cf-PWV) to calculate the propagation time of the arterial pressure waveform as it travels to the carotid to femoral arteries from the heart [15, 16]. It is considered a reliable non-invasive technique to study arterial stiffness and is used as a cardiac-risk stratification marker among healthy and a wide range of patient populations [17–19]. However, its utility beyond the assessment of arterial stiffness for cardiovascular risk assessment (e.g., using HRV) has never been investigated before. Therefore, a reliability study is warranted to examine the reproducibility and consistency of this new method (cf-PWV) to measure HRV. We anticipate that establishing the reliability of cf-PWV for HRV_UST_ measurement would add to the current use of the Vicorder device i.e. PWV measurement.

Therefore, in this study, we sought to compare the influence of signal acquisition sites (carotid vs femoral arteries) on the reliability of HRV_UST_ measurement and secondly the inter-rater agreement in the preferable site. We hypothesised that femoral waveforms would provide sharper and more reliable signals to derive HRV_UST_ and offer good inter-rater reliability.

## Materials and methods

### Study design

This was an inter-rater reliability study in which the two raters used the same method, instrument, and procedure to assess the same subjects [20]. The present study was reported in accordance with the Guidelines for Reporting Reliability and Agreement Studies [20] (S1 Table). We used the baseline data from the ArmeD SerVices TrAuma and RehabilitatioN OutComE (ADVANCE) prospective cohort study [21].

### Participants

Given the paucity of evidence on HRV in people with non-acute traumatic injuries [22], we randomly selected a sample of 50 injured adult male participants recruited into the ongoing ADVANCE study [21]. The ADVANCE study is investigating the long-term physical and psycho-social outcomes of male battlefield casualties from the UK Armed Forces following deployment to Afghanistan (2003–2014) [21]. The full protocol of the ADVANCE study has been recently published [21]. It has been approved by the Ministry of Defence Research and Ethics Committee (MoDREC) (protocol number 357/PPE/12) and is conducted in compliance with the Declaration of Helsinki [21]. In compliance with data confidentiality, authors had no access to any information regarding participants’ individual identity during and after data collection. All data were only accessible to authorised authors (RM, CB, and ANB). However, the minimal dataset can be found in the supporting information (S2 Table).

The participants were included in the injured group if they had sustained a physical combat injury during their deployment to Afghanistan (2003–2014) that required an aeromedical evacuation to a UK hospital for treatment. Participants with a history of cardiovascular diseases or acute medical conditions were excluded [21]. Patient Health Questionnaire (PHQ-9) was used as one of the measures for mental health (depression) [23]. None of the participants was on oral rate-controlling medicines such as beta-blockers or rate-limiting calcium channel blockers.

All participants gave informed written consent after reading the protocol in the presence of a trained research nurse. The data were collected between August 2015 and August 2020 and were accessed for the present study in March 2022. All assessments were conducted by a trained research nurse at Defence Medical Rehabilitation Centre (DMRC), Stanford Hall, Loughborough [21].

### Procedure

The participants avoided food and caffeine for ≥8 hours before the procedure. PWV was measured using the Vicorder device (Skidmore Medical Limited, Bristol, UK) which is an inflatable cuff-based device that simultaneously measures the upstroke of carotid and femoral pulsations to calculate PWV [21]. PWV measurements took place in a temperature-controlled room on a hospital bed with participants in the supine position with their head raised to 30° in order to maintain the relaxation of the skin and muscles overlying the carotid artery. The participants were encouraged to relax, breathe normally, and avoid talking or sleeping during the procedure.

A 30‐mm plethysmographic partial inflatable sensor was placed over the neck (carotid artery) and a 100‐mm wide BP cuff was placed over the left thigh (femoral artery) [21]. Carotid and femoral arterial waveforms were sampled simultaneously during PWV and in sequential triplicates [21]. All recordings were taken ipsilaterally unless impossible due to amputation (alternatively contralateral recordings). The Vicorder records a continuous arterial waveform measurement for IBI calculation for a period of up to 16 seconds which was used to measure HRV_UST_. All data were stored on the Vicorder database.

### Data processing and analysis

The data were then exported into Microsoft Excel for processing. To begin offline HRV analysis, the processed data were imported into Kubios Premium (V. 3.2, The Biomedical Signals Analysis and Medical Imaging Group, University of Kuopio, Finland). As a primary outcome measure, only the time domain HRV measure of RMSSD (root mean square of successive differences) was calculated in this study given its established reliability and validity in HRV_UST_ analysis [4–6].

All arterial waveform data were visually inspected to locate any ectopic, missed, or misplaced beats, and were manually corrected when needed. For data processing in Kubios Premium, the smoothness priors (500), and interpolation (cubic spline: 4 Hz with 50ms R-R threshold) were chosen as previously used [24]. The maximal acceleration point in the upslope in the arterial waveform was the default selection within Kubios to determine the IBI as previously validated [24]. The automatic correction and noise levels were set to none and medium, respectively throughout the analysis. Since the minimum analysable length of the signal in Kubios is 10 seconds, the signals shorter than 10 seconds were interpolated using the merge analysis type. Otherwise, the analysis type was set to single.

### Reliability protocol

In order to determine which of these two sites (carotid vs femoral) was preferable for HRV_UST_ analysis, firstly, their signal quality was compared in paired data points by a single rater (RM). Based on signal quality, thereafter, the blinded inter-rater reliability for the preference site (carotid vs femoral) was examined by measuring the paired correlation and agreement in their RMSSD data by two raters (RM and CJB) using an identical protocol, independently. Rater 1 (RM) had received preliminary training in the analysis of HRV using Kubios whereas Rater 2 (CJB) had more experience in HRV analysis.

### Statistical analysis

**Fig 1:**
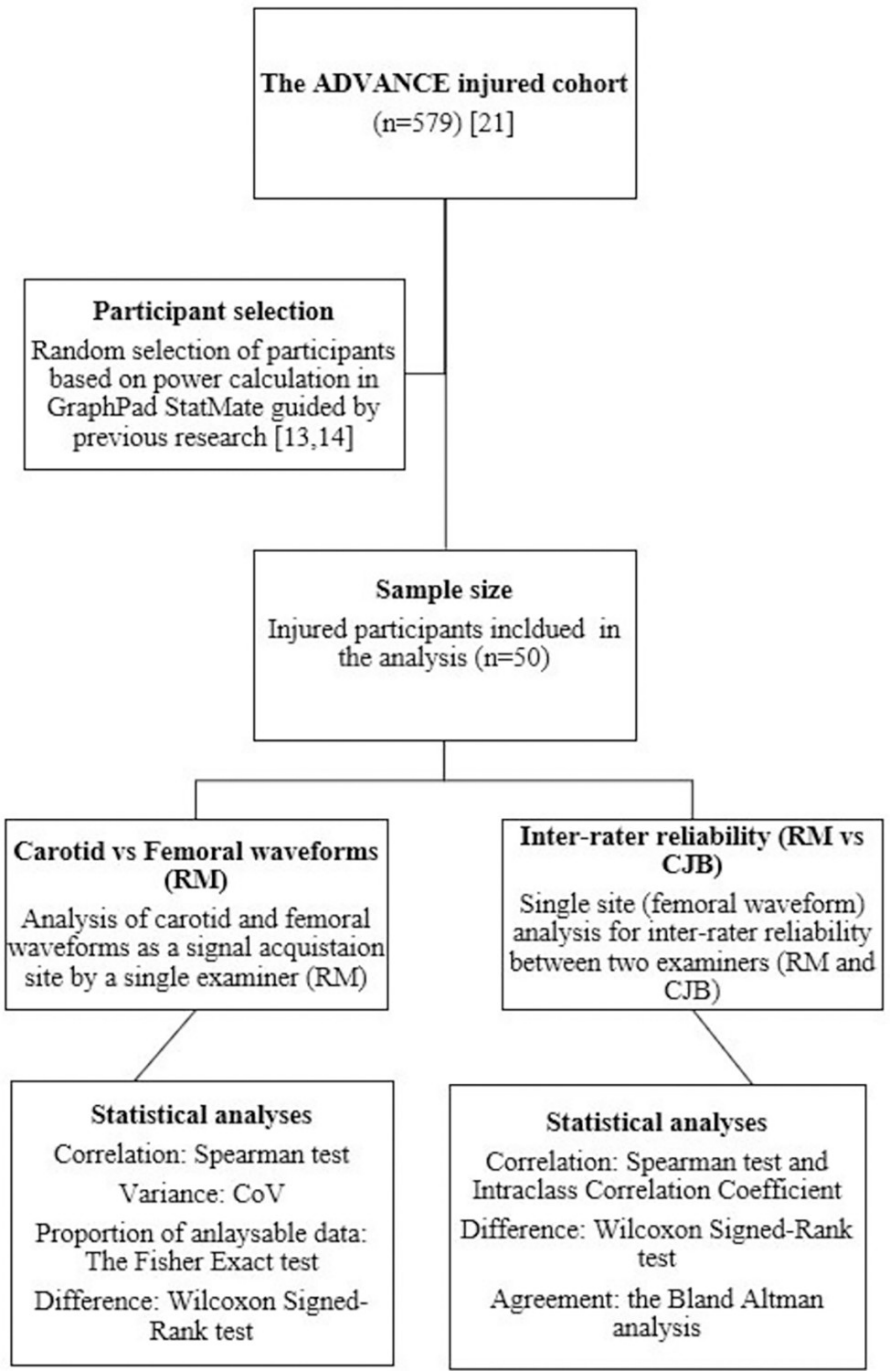
Summary of statistical analyses.

The normality of continuous data was assessed by inspecting their frequency histograms and using the Shapiro-Wilk test. The results of continuous data are presented as mean ± standard deviation (SD) or 95% confidence interval (CI) for normally distributed data and as the median and interquartile range (IQR) for skewed data. A summary of the statistical analyses is shown in Fig 1.

The Fisher Exact test was used to determine the relative proportion of analysable data provided by the carotid vs femoral sites. The variation within single-rater repeated RMSDD testing for the carotid and femoral arterial sites was quantified using the Coefficient of Variance (CoV) and 95% CI for the paired data points. The RMSSD data qualified for non-parametric tests. Differences in paired continuous RMSSD data were quantified using the Wilcoxon-Signed Rank test. Their correlations were examined using the Spearman correlation with 95% CI and interpreted as weak (0.10.-0.39), moderate (0.40–0.69), strong (0.70–0.89), and very strong (0.90–1.00) as previously described [25]. The inter-rater agreement in RMSSD scores (femoral only) was examined using the Bland-Altman analysis in which the average RMSSD (x-axis) was compared with the differences in RMSSD values obtained by two raters (y-axis). The average difference (bias) and 95% CI Limits of Agreement (LoA) were reported as results. A two-tailed p-value < 0.05 was set as the level of significance. These analyses were conducted in Stata (V 15.0; StataCorp LLC) unless otherwise stated.

The inter-rater reliability of HRV_UST_ between the two raters (RM and CJB) was further determined by calculating the Intra-class Correlation Coefficient (ICC) as previously used [24, 26, 27] and recommended [28]. The absolute ICC was calculated in SPSS (V 28.0.1.1; IBM SPSS statistics). The selection of the model- two-way mixed effect- was guided by previous literature [28, 29]. An absolute ICC score of 0.90–1.00, 0.50–0.75, <0.5 was considered to be an excellent, moderate, and poor score of inter-rater reliability, respectively [29].

### Sample size calculation

Whilst there are published reference and comparative values for 10-s vs 1–5-minute ECG-derived RMSSD data [4, 6, 13, 14, 30], there is a paucity of paired validation data related to 10-s HRV using non-ECG-derived signals to generate HRV. In a previous study of 12 healthy military servicemen, the authors observed a strong correlation between paired digital-pulse derived (ithlete^TM^) 55-s RMSSD scores (Spearman r = 0.89; 95% CI 0.82–0.93), although the mean difference was not reported [13]. Using this data and our own pilot data [13, 14], we calculated that a sample size of 50 paired data would provide ≥80% power to detect an average difference in RMSSD means of ≥1.60ms with a significance level (alpha) of 0.05 (two-tailed). The sample size calculations were performed in GraphPad StatMate (V 2.00).

## Results

The mean age of participants at the time of injury was 27.08±4.91 (range 18–27) years and at assessment was 34.06±4.88 (range 23–44) years. Of 50 participants, 27 were non-amputees (54%) and 23 were amputees (46%). Most of the participants were White British (92%) on lower rank at the time of sampling (68%), and never smoked (46%). Participants scored a median of 2.5 (Interquartile range: 1, 9) on PHQ-9, reflecting relatively good mental health.

### Carotid vs femoral waveforms

**Fig 2:**
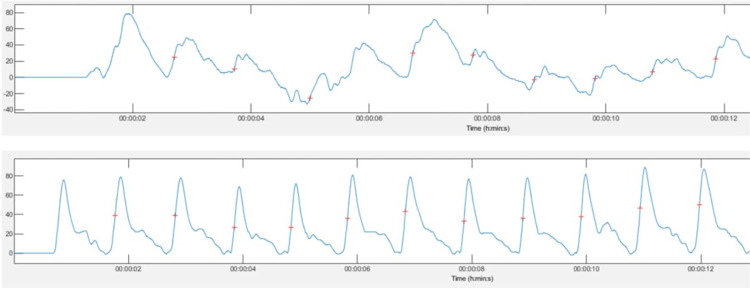
Comparison between carotid (top) and femoral (bottom) waveforms as analysed in Kubios (V. 3.2, The Biomedical Signals Analysis and Medical Imaging Group, University of Kuopio, Finland).

The Fisher Exact test showed that 63.8% (n = 88/138) of the available carotid waveforms were of optimal quality compared with 96.3% (n = 130/135) of the femoral waveforms examined (p<0.001). The femoral waveforms were superior to the carotid waveforms as they provided clearer and sharper signals (Fig 2).

There were 76 available paired carotid and femoral data points that were used to compare their relative HRV_UST_ reliability and agreement. Of these, the CoV of triplicate RMSSD measurements were non-significantly lower using the femoral (26.59±16.51%, 95%CI 20.73–32.44) compared with the carotid waveforms (28.47±17.05%, 95%CI 22.42–34.51). RMSSD scores were significantly higher from the carotid artery compared with the femoral artery with a significant median difference of 4.70ms (IQR: -0.58, 12.34, p<0.001). There was a strong, correlation between RMSSD measured from the carotid and femoral waveforms (r = 0.82, p<0.001) (Table 1).

**Table 1:** Comparison of single-rater reliability for carotid and femoral HRV_UST_ and the inter-rater reliability for femoral HRV_UST_.

Single rater reliability (Carotid vs Femoral)	Carotid	Femoral	Difference (Carotid-Femoral) (Median, IQR or Mean, SD)	P value†	Spearman Correlation coefficient (95% CI)	P value†
Paired data points	76	76	-	-	-	-
RMSSD, ms	37.20 (25.06, 48.75)	31.43 (20.76, 40.77)	4.70 (-0.58, 12.34)	<0.001	0.82 (0.73–0.88)	<0.001
CoV, %	28.47±17.05 (22.42, 34.51)	26.59±16.51 (20.73, 32.44)	1.88±20.04 (0.52, 8.98)	0.85	-	-
**Inter-rater reliability (Femoral)**	**Femoral (CJB)**	**Femoral (RM)**	**Difference (Rater 1–2) (Median, IQR)**	**P value†**	**Spearman Correlation Coefficient (95% CI)**	**P value†**
Paired data points	130	130	-	-	-	-
RMSSD, ms	33.97 (25.43, 49.15)	32.93 (24.96, 48.73)	0.0 (-0.28, 0.03)	0.20	0.97 (0.54–0.94)	<0.001

Data presented as mean ± SD or number (%) or median (IQR) for skewed data. Differences and correlations are presented as median (IQR) or mean (SD) and rho with 95%CI respectively

RMSSD, Root Mean Square of Successive Differences; CoV, Coefficient of Variation

† Appropriate equality test based on normality

### Inter-rater reliability for femoral HRV

**Fig 3:**
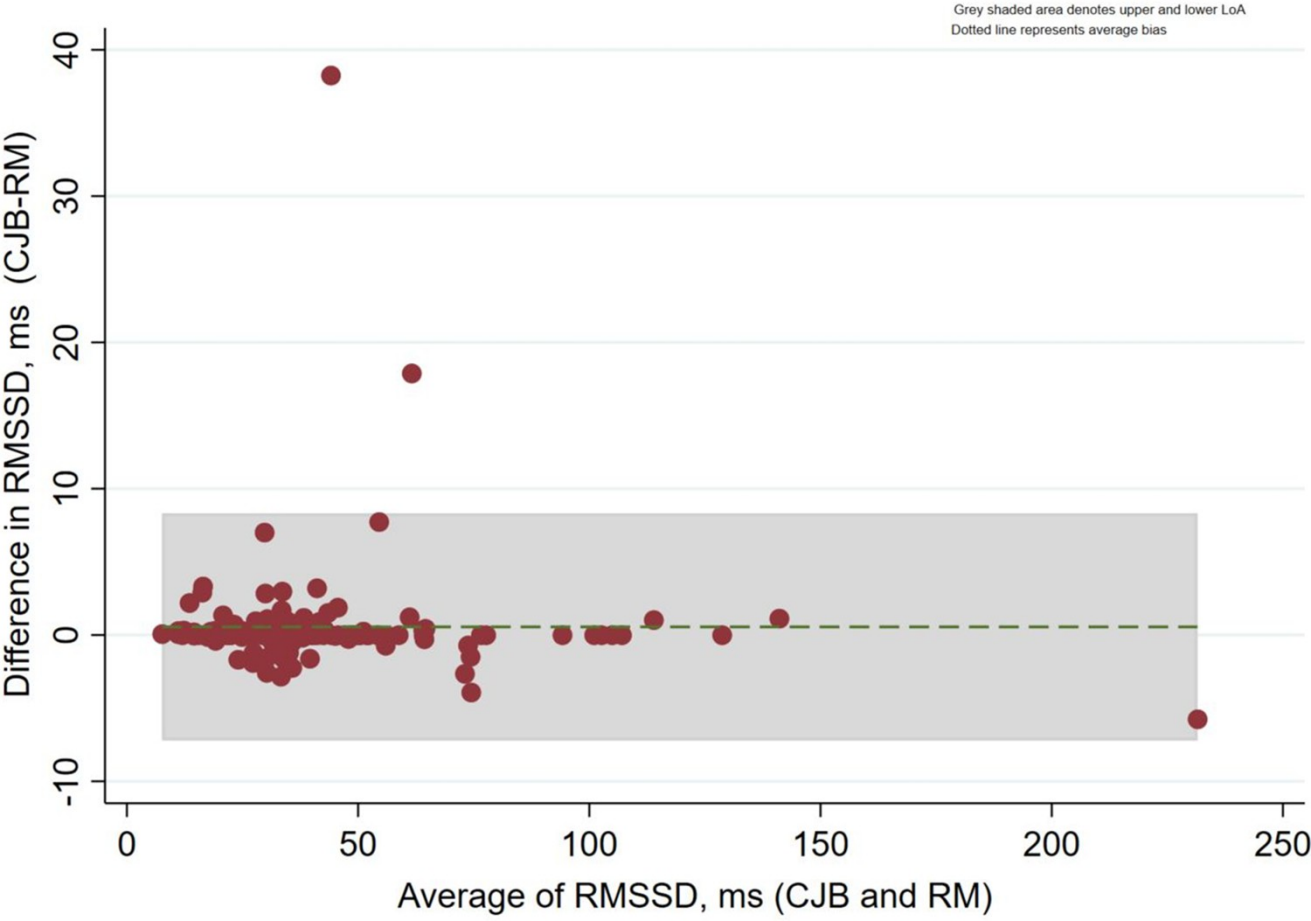
The Bland-Altman plot comparing the difference in RMSSD (ms) values obtained by two examiners (RM and CJB), the limits of agreement (±LoA), and their 95% CI.

There were 130 RMSSD paired data examined for inter-rater reliability between the two raters (RM and CJB). The median difference in RMSSD recorded by RM and CJB was statistically insignificant (p = 0.20) (Table 1). A very strong correlation between RMSSD measured by RM and CJB (n = 130, r = 0.97, p<0.001) was also reflected in the Intraclass Correlation Coefficient [ICC 0.99 (95%CI: 0.994–0.997)]. On Bland-Altman analysis, there was a very strong agreement in RMSSD values obtained by RM and CJB with a median difference of 0.0ms (IQR; -0.28, 0.03) and a mean difference of 0.55ms, (LoA -7.21 to 8.32ms) with 98.5% (128/130) of all paired RMSSD values falling within 95%CI LoA (Fig 3).

## Discussion

To the authors’ knowledge, this is the first study to investigate the reliability of signal acquisition sites (carotid and femoral arteries) as a proxy source for HRV_UST_ measurement and determine the inter-rater reliability in a subset of the ADVANCE injured cohort. The findings suggest that the femoral waveform measured using the Vicorder device can be used to measure HRV_UST_ (RMSSD), offering excellent inter-rater reliability (agreement) between the two raters.

HRV_UST_ offers great potential to be used in clinical settings given its time-effectiveness. Given the rapid increase in use of wearable devices, application of HRV_UST_ may be clinically relevant especially in daily tracking and monitoring HRV; however, this warrants validation of accuracy and predictive power of HRV_UST_ [31]. One such validated measure of HRV_UST_, as used in this study, is RMSSD which appears to be the most consistently reported HRV_UST_ measurement [5]. The reason for this is its relative resilience to the effects of shorter recording periods and the influence of respiration [3, 5]. RMSSD is strongly correlated with high-frequency (HF) power and depicts vagally mediated cardiac control and parasympathetic activity [3].

The data from our study has shown that the femoral waveforms provided a greater proportion of analysable data and clearer signals for HRV_UST_ measurement (RMSSD) than that of carotid waveform and should be preferably used for HRV_UST_ analysis. This finding agrees with those reported by Parikh and colleagues [32]. They also reported that the carotid waveforms were more frequently suboptimal than that of the femoral artery [32]. Carotid-femoral PWV requires a high level of technical precision during measurement [19]. A plausible explanation for the relatively poorer signal quality from the carotid waveform may relate to patient compliance with the neck cuff [19] and the influence of respiratory movement [33] or jugular vein interference as recently reported [34]. The femoral artery is more peripheral and less elastic than the carotid. Consequently, it is subject to greater pressure amplification and a higher and more defined waveform peak, which is likely to be advantageous for the measurement of the cardiac IBI [35, 36]. Conversely, it is a more intrusive site to examine. Our data would suggest that in the event where an analysable signal is unobtainable from the femoral waveform and the carotid waveform trace is good, the carotid waveform can be used instead given their significant correlation observed in the present study. It is our personnel recommendation that the reading with the greatest number of beats should be used for HRV_UST_ measurement.

One of the limitations of the available PWA-derived HRV_UST_ has been the inability to manually exclude ectopic or artefactual beats. This is crucial as even a single ectopic or artefactual beat can markedly influence HRV_UST_ given the very short recording duration [14]. In our study, we were able to manually review the arterial IBI data prior to HRV analysis which offered a potential advantage over the aforementioned devices. The fact that we sampled a more proximal large site in the femoral versus the brachial (e.g., Uscom BP^+^), radial (e.g., SphygmoCor), or digital artery (e.g., ithlete^TM^) may be advantageous given its excellent signal quality and proximity to the heart.

Whilst the time domain measures of HRV_UST_ have been shown to strongly agree with those of longer HRV recordings of up to 5-minutes, the validation data has been largely focussed on ECG rather than PWA-derived data and supports its use as a valid HRV measurement tool [4, 6, 37]. It has been reported that a CoV of <30% can be considered an acceptable reliability target for repeatability testing for time-domain measures of short-term HRV [37]. However, no such cut-off is available for HRV_UST._ It has been well-shown that the degree of repeated measure agreement in HRV decreases as the recording period shortens. Despite this, the CoV for repeated testing in both the femoral and carotid arteries were both <30% at 26.59% and 28.47% respectively. Potential reasons for this imperfect agreement could plausibly relate to our study population of injured adults. Finally, the default criteria, for the selection of the fiducial point for IBI calculation, can differ between HRV software manufacturers [38] with some using the arterial waveform peak or nadir rather than maximal acceleration (Kubios) within the arterial waveform.

Key strengths of this study include the novelty of the HRV_UST_ measurement method using cf-PWV and the conduct of blinded inter-rater measurements of RMSSD. It is also, to the author’s knowledge inclusion of the first study to examine HRV_UST_ following non-acute CRTI. Unlike other reliability studies conducted in this field [26, 27], this study demonstrates statistical rigor with the inclusion of a wide range of statistical tests i.e., CoV, Spearman correlation, Wilcoxon Signed-Rank test, ICC, and the Bland-Altman plot. We have also interpreted the results in accordance with the recent recommendations on HRV_UST_ analysis [1] and reported the study following the Guidelines for Reporting Reliability and Agreement Studies [20]. Our findings met the criterion proposed by Pecchia and colleagues i.e., a good correlation (compared with a given threshold), non-biasness, and statistically significant agreement between the two methods determine the reliability of HRV_UST_ as a surrogate marker [1].

There are a few limitations that should be acknowledged. Firstly, for the true assessment of the sympathetic nervous system, the use of carotid-femoral waveform has been questioned [39] and instead the assessment of QT variability has been recommended [40]. Nevertheless, we reported RMSSD- a conventional marker of the parasympathetic system- in an attempt to minimise this issue. Further to this, given the increase in sympatho-vagal (as a measure of Low frequency/High frequency ratio) balance in response to non-acute injury [22], we do not suspect any changes in the level of agreement introduced by alternations in sympatho-vagal balance. Secondly, our sample size was relatively small, and we only included male participants with physical combat injuries. Lastly, a comparison of our Vicorder cf-PWV-derived HRV_UST_ to that using traditional ECG-derived HRV via a validity study could not be performed as this data was not available at the baseline level of the ADVANCE study. However, we plan to examine this in the future at the first follow-up (three years after baseline). Other limitations include the lack of a control group and analysis of only one time-domain HRV measure (RMSSD).

Nonetheless, our results have shown that the Vicorder signal can be used as an untraditional source of HRV data in addition to its primary use i.e., PWV measurement. The ability to provide additional HRV data over a very short time period enhances its clinical applicability. This has potential translational applications whereby devices such as the Vicorder, could provide supplementary cardiovascular risk data. While we have investigated the reliability of cf-PWV using a validated measure of HRV_UST_ (RMSSD), it might of interest to employ other approaches of ultra-short-term analysis such as symbolic analysis [41] in the future to extend the reliability of the proposed method. Similarly, researchers may also replicate our proposed technique in different population to extend and verify the reliability of cf-PWV, with perhaps a comparison with brachial-femoral PWV, for HRV measurement. Overall, this holds immense potential as a combination cardiovascular risk marker, especially in those settings where the analysis of ECG signals is hindered owing to noise, or traditional acquisition of ECG data is impossible such as triage during military operations and evacuation [42, 43].

## Conclusion

This study described a novel method to measure HRV_UST_ using the carotid-femoral waveforms obtained from the pulse wave velocity in a cohort of male battlefield casualties from the UK Armed Forces. The findings support our hypothesis that femoral arterial waveform may provide a reliable proxy source of HRV_UST_ measurement, offering excellent inter-rater reliability. Owing to greater signal clarity, the femoral waveform would be preferable over the carotid waveform to derive HRV_UST_. More longitudinal studies are needed to investigate this further with a large sample size and a control group to understand the impact of physical injuries on HRV_UST_ measures including in military populations.

## Supporting information

S1 Table The guidelines for reporting reliability and agreement studies (GRRAS) checklist. (PDF)

S2 Table Minimal dataset used in the current study. (PDF)
