# Supplementary material for: Reliability of carotid-femoral arterial waveforms for the derivation of ultra-short term heart rate variability in injured British servicemen: An inter-rater reliability study
Source: PLoS One. 2023 Sep 1;18(9):e0290618. doi: 10.1371/journal.pone.0290618 (PMC10473479; doi:10.1371/journal.pone.0290618)
Supplement: S2 Table — (PDF) [file pone.0290618.s002.pdf]

S2 Table: Minimal dataset used in the current study

For Carotid vs Femoral				For inter-rater reliability using Femoral waveform (Rater 1 vs Rater 2)			
Paired data point	RMSSD from Carotid	RMSSD from Femoral	Difference in RMSSD (Carotid-Femoral)	Paired data point	RMSSD_Rater 1	RMSSD_Rater 2	Difference in RMSSD (Rater 1- Rater 2)
1	40.525	24.727	15.798	1	24.727	25.014	-0.287
2	29.885	32.596	-2.711	2	33.979	33.979	0
3	49.434	24.517	24.917	3	27.489	27.63	-0.141
4	23.321	17.893	5.428	4	32.596	32.596	0
5	16.886	12.112	4.774	5	24.517	24.517	0
6	20.345	25.434	-5.089	6	17.893	17.893	0
7	27.358	14.812	12.546	7	12.112	12.43	-0.318
8	19.536	14.521	5.015	8	140.5	141.63	-1.13
9	81.261	50.079	31.182	9	25.434	25.434	0
10	23.817	24.91	-1.093	10	14.812	17.72	-2.908
11	41.753	56.66	-14.907	11	12.414	14.61	-2.196
12	73.769	74.382	-0.613	12	14.521	14.49	0.031
13	42.079	32.341	9.738	13	24.965	63.22	-38.255
14	51.627	50.301	1.326	14	27.276	27.16	0.116
15	34.18	31.934	2.246	15	30.365	30.37	-0.005
16	49.08	40.349	8.731	16	50.079	50.079	0
17	28.406	27.351	1.055	17	24.91	24.79	0.12
18	92.274	42.448	49.826	18	94.159	94.159	0
19	94.311	47.997	46.314	19	56.66	56.59	0.07
20	56.949	29.954	26.995	20	74.382	71.74	2.642
21	48.833	52.631	-3.798	21	32.341	31.88	0.461
22	26.453	25.94	0.513	22	50.301	50.28	0.021
23	62.905	76.516	-13.611	23	30.333	30.333	0
24	46.428	33.275	13.153	24	32.377	32.311	0.066
25	62.85	64.432	-1.582	25	52.04	52.04	0
26	52.231	52.048	0.183	26	104.9	104.9	0
27	34.058	41.126	-7.068	27	45.036	44.985	0.051
28	39.926	31.368	8.558	28	31.934	31.934	0
29	40.539	30.21	10.329	29	22.387	22.387	0
30	20.856	15.62	5.236	30	40.349	38.74	1.609

31	25.014	11.973	13.041	31	27.351	28.31	-0.959
32	51.845	31.49	20.355	32	50.995	51.24	-0.245
33	15.958	11.006	4.952	33	39.095	39.095	0
34	13.533	18.718	-5.185	34	42.448	42.448	0
35	12.612	27.037	-14.425	35	47.997	47.74	0.257
36	30.911	27.628	3.283	36	29.954	29.954	0
37	30.814	34.684	-3.87	37	52.631	70.52	-17.889
38	48.686	39.47	9.216	38	107.01	107.01	0
39	46.809	38.057	8.752	39	25.94	25.94	0
40	60.485	48.347	12.138	40	37.612	37.612	0
41	19.618	10.924	8.694	41	32.313	32.313	0
42	35.423	22.772	12.651	42	29.77	29.94	-0.17
43	45.993	35.857	10.136	43	30.94	29.37	1.57
44	37.089	35.716	1.373	44	50.658	58.39	-7.732
45	48.557	44.646	3.911	45	76.516	76.516	0
46	30.99	17.477	13.513	46	33.275	34.39	-1.115
47	84.296	76.389	7.907	47	64.432	64.16	0.272
48	46.6	32.572	14.028	48	52.048	52.03	0.018
49	15.345	28.492	-13.147	49	41.126	41.13	-0.004
50	37.318	34.586	2.732	50	31.368	31.014	0.354
51	72.697	77.656	-4.959	51	30.21	31.05	-0.84
52	19.52	14.357	5.163	52	15.62	15.63	-0.01
53	40.96	44.187	-3.227	53	113.4	114.43	-1.03
54	25.425	24.887	0.538	54	11.973	11.973	0
55	20.747	20.075	0.672	55	31.49	28.92	2.57
56	29.181	21.448	7.733	56	11.006	11.08	-0.074
57	28.43	22.457	5.973	57	30.628	30.722	-0.094
58	19.111	17.605	1.506	58	42.472	42.84	-0.368
59	16.706	17.253	-0.547	59	18.718	19.006	-0.288
60	36.602	19.218	17.384	60	27.037	27.037	0
61	37.056	33.977	3.079	61	27.628	27.93	-0.302
62	23.608	7.5578	16.0502	62	29.9	29.9	0
63	23.043	19.376	3.667	63	64.305	64.74	-0.435
64	43.646	49.157	-5.511	64	34.684	31.85	2.834
65	25.122	19.333	5.789	65	39.47	42.677	-3.207

66	49.288	45.644	3.644	66	38.057	37.893	0.164
67	41.863	28.189	13.674	67	41.142	42.05	-0.908
68	41.47	36.827	4.643	68	38.952	38.952	0
69	44.745	26.238	18.507	69	64.118	64.118	0
70	41.986	39.775	2.211	70	60.489	61.71	-1.221
71	57.691	40.424	17.267	71	48.347	48.347	0
72	69.764	35.853	33.911	72	101.01	101.01	0
73	28.342	29.791	-1.449	73	34.325	35.252	-0.927
74	18.51	17.945	0.565	74	10.924	11.2	-0.276
75	30.579	32.115	-1.536	75	18.741	18.741	0
76	44.284	47.087	-2.803	76	22.772	23.493	-0.721
				77	35.857	35.34	0.517
				78	35.716	35.161	0.555
				79	42.692	44.212	-1.52
				80	44.646	46.53	-1.884
				81	17.477	17.36	0.117
				82	76.389	72.47	3.919
				83	102.63	102.63	0
				84	32.572	34.267	-1.695
				85	14.763	18.09	-3.327
				86	28.492	31.339	-2.847
				87	29.333	29.33	0.003
				88	34.586	33	1.586
				89	77.656	77.656	0
				90	14.357	14.554	-0.197
				91	44.187	44.187	0
				92	24.887	23.21	1.677
				93	20.075	21.43	-1.355
				94	21.448	21.47	-0.022
				95	22.457	22.457	0
				96	17.605	17.605	0
				97	75.022	73.54	1.482
				98	17.253	17.253	0
				99	21.691	21.691	0
				100	35.618	34.4	1.218

				101	26.076	26.05	0.026
				102	19.218	19.218	0
				103	33.977	33.977	0
				104	7.5578	7.63	-0.0722
				105	54.165	54.165	0
				106	63.894	64.22	-0.326
				107	19.376	19.376	0
				108	48.738	48.738	0
				109	58.743	58.743	0
				110	49.157	49.157	0
				111	128.69	128.69	0
				112	37.636	38.82	-1.184
				113	19.333	18.95	0.383
				114	45.644	45.644	0
				115	28.189	26.288	1.901
				116	36.827	34.59	2.237
				117	26.238	33.252	-7.014
				118	39.775	39.775	0
				119	74.106	73.401	0.705
				120	40.424	40.424	0
				121	35.853	35.853	0
				122	56.287	55.57	0.717
				123	234.39	228.64	5.75
				124	29.791	30.89	-1.099
				125	32.102	35.08	-2.978
				126	17.945	18.18	-0.235
				127	21.78	22.55	-0.77
				128	27.938	26.73	1.208
				129	32.115	30.89	1.225
				130	47.087	47.087	0
